# Making the library accessible for all: a practical guide for librarians

**DOI:** 10.29173/jchla29880

**Published:** 2025-08-01

**Authors:** Hailey Siracky

**Affiliations:** Faculty Librarian Mount Royal University Library Calgary, AB, Canada

Vincent J. **Making the library accessible for all: a practical guide for librarians**. 2nd ed. Lanham, MD: Rowman & Littlefield; 2024. Softcover: 202 p. ISBN: 978-1-5381-7681-8. Price: USD$65.00. Available from: https://www.bloomsbury.com/us/making-the-library-accessible-for-all-9781538176818/.

In recent years, conversations around accessibility in libraries have deepened and expanded, inviting a reevaluation of access, equity, and inclusion efforts grounded in lived experience and contemporary disability justice. *Making the library accessible for all: a practical guide for librarians* (second edition), by Jane Vincent, positions itself as a practical resource for library professionals seeking to improve access across library services and spaces.

Author Jane Vincent is the assistive technology manager at the University of Michigan and holds an MA in Library and Information Science from the same institution. This text is an updated version of her original 2013 edition. Changes to the text include expanding the scope of content to include academic, public, and special libraries, updating terminology to align more closely with current best practices, and incorporating information on new legislation and standards related to accessibility, such as the Marrakesh Treaty [1], an international agreement that enables countries to create and share books in accessible formats for people who are blind, visually impaired, or otherwise print-disabled.

Additionally—and perhaps most significantly—the second edition incorporates interviews with librarians and accessibility experts, many of whom identify as disabled. These first-person insights foreground lived experience and offer critical context for understanding both the stakes and the impact of accessibility work. The book consists of eight chapters structured around practical domains of library service, such as communications, materials, architecture, events, and technology, as well as a preface that articulates the book’s rationale for improving accessibility in library spaces—a section that, crucially, frames the reader’s understanding of what access work entails.

While *Making the library accessible for all* has deservedly received praise for its usefulness and actionability, and goes on to offer abundant practical and care-oriented information on improving accessibility across many domains of library work, it opens with a framing that raises concerns for me as a neurodivergent librarian and researcher. In this opening preface, accessibility is presented not as an ethical obligation or a matter of rights, but as a means for institutional benefit: a way to attract new patrons, improve reputation, and increase enthusiasm about the library among the public.

The early framing of disabled patrons as objects of service or institutional gain is especially troubling given how overt it is. The book’s opening paragraph states: “Your library still needs and wants to attract more patrons with disabilities” [2]. This phrasing positions disabled people as external to the library’s core community—individuals to be drawn in for institutional benefit rather than recognized as part of the library’s constituency. Later, a section titled “Impetus” attempts to make the case for improving accessibility through a story about a planter that obstructs a walkway, and that also happens to be a source of irritation for library employees. In the story, the planter gets moved, and the benefits are expressed this way: “Your library immediately becomes more ADA compliant. Fewer shins are bruised. Everyone is happy” [3]. This framing equates mild physical inconvenience with the systemic exclusion faced by disabled patrons, flattening life-limiting barriers into a shared irritation and arguably still prioritizing nondisabled discomfort.

Elsewhere in the preface, the author suggests inviting disabled patrons to lead film discussions at disability-themed events in order to expose the rest of the community to “novel ideas and perspectives” [4]. This not only assumes disabled patrons are not already part of the community, it also casts them primarily as educational content or entertainment for a nondisabled audience. In another example, librarians are encouraged to collect affidavits of support from the library’s wheelchair users to help lobby for a new bus stop in front of the library, positioning disabled patrons as instruments for institutional advocacy rather than as stakeholders in the outcome. In both cases, disabled people are framed not in terms of access, inclusion, or equity, but by how effectively they can enhance the library’s image, programming, or political leverage. This opening chapter undermines the seriousness of the subject matter at hand, and introduces a transactional logic that casts the remainder of the book—which is deeply knowledgeable, nuanced, and caring—with a shadow it doesn’t deserve, and that took this reader considerable effort to see beyond.

After a troubling preface, the remainder of *Making the library accessible for all* diverges significantly in tone and appears more closely aligned with contemporary disability advocacy. The content of the main text feels sincere and justice-oriented, making the early framing all the more jarring. In terms of practical content, this text offers an abundance of information that is specific to library contexts and grounded in the author’s extensive experience as an accessibility technologist. Recommendations move beyond compliance and institutional optics, emphasizing respect, specificity, and a genuine attentiveness to disabled people’s lived experiences. The book affirms that accessibility work must be led by those directly affected, encourages readers to compensate disabled people for their insight, and explicitly discourages generalized or monolithic representations of disability. Its treatment of person-first versus identity-first language is particularly nuanced, aligning with contemporary advocacy in a way that feels both informed and respectful. The author highlights intersectionality, acknowledges the reality of conflicting access needs, and resists the common framing that “access benefits everyone” should be the primary justification for change, noting that while this may be true, accessibility efforts are meant to serve a specific population whose needs must be prioritized. The values of respect, specificity, and accountability to disabled individuals remain consistent throughout the remainder of the book, in ways that sharply contrast with the preface—and that make its earlier missteps feel less like the book’s core ethic and more like an unrepresentative framing error.

Beyond its tone and values, the book’s structure and supporting materials contribute significantly to its usefulness. Designed as part of the *Practical Guide for Librarians* series, it offers clearly organized chapters that make it easy to consult for specific questions or use cases, making it particularly well suited to use as a reference tool. Appendices include practical checklists (such as an especially helpful one for accessible presentation slide design), a glossary, and a list of abbreviations, all of which contribute to its digestibility for time-pressed readers. Though its legal references are based on American legislation, including the Americans with Disabilities Act and Section 508, Canadian readers may still find these discussions informative in illustrating how accessibility is framed in disability law more broadly. Overall, the book’s organization and wealth of practical tools add to its value for library staff looking to improve accessibility in concrete, actionable ways.

While *Making the library accessible for all* opens on uncertain footing, what follows is thoughtful, well-researched, and grounded in lived experience. The inclusion of disabled voices, the clear engagement with current advocacy, and the wealth of practical tools all contribute to a resource that many library workers will find genuinely useful. Readers should approach the book with a critical eye—particularly in regard to its opening framing—but will likely find that chapters offer concrete, inclusive, and justice-oriented guidance. As a reference tool for accessibility work across different domains of library service, this book has real value, especially when supplemented by additional resources that foreground structural accountability and community leadership.
